# Cognitive testing of physical activity and acculturation questions in recent and long-term Latino immigrants

**DOI:** 10.1186/1471-2458-10-481

**Published:** 2010-08-13

**Authors:** David Berrigan, Barbara H Forsyth, Cynthia Helba, Kerry Levin, Alicia Norberg, Gordon B Willis

**Affiliations:** 1 Applied Research Program, Division of Cancer Control and Population Sciences, National Cancer Institute, Executive Plaza North MSC 7344, Bethesda MD 20892-7344, USA; 2 Center for Advanced Study of Language, University of Maryland, Box 25, College Park, MD 20742-0025, USA; 3 Westat, 1650 Research Boulevard, Rockville, MD 20850, USA

## Abstract

**Background:**

We ascertained the degree to which language (English versus Spanish), and residence time in the US influence responses to survey questions concerning two topics: self-reported acculturation status, and recent physical activity (PA). This topic is likely to be of general interest because of growing numbers of immigrants in countries worldwide.

**Methods:**

We carried out qualitative (cognitive) interviews of survey items on acculturation and physical activity on 27 Latino subjects from three groups: (a) In Spanish, of those of low residence time (less than five years living in the U.S.) (n = 9); (b) In Spanish, of those of high residence time (15 or more years in the U.S) (n = 9); and (c) in English, of those of high residence time (n = 9).

**Results:**

There were very few language translation problems; general question design defects and socio-cultural challenges to survey responses were more common. Problems were found for both acculturation and PA questions, with distinct problem types for the two question areas. Residence time/language group was weakly associated with overall frequency of problems observed: low residence time/Spanish (86%), high residence time/Spanish (67%), and English speaking groups (62%).

**Conclusions:**

Standardized survey questions related to acculturation and physical activity present somewhat different cognitive challenges. For PA related questions, problems with such questions were similar regardless of subject residence time or language preference. For acculturation related questions, residence time/language or education level influenced responses to such questions. These observations should help in the interpretation of survey results for culturally diverse populations.

## Background

A 2008 estimate indicates that the US population includes over 46 million Latinos or approximately 15.4% of the total population http://factfinder.census.gov/ and that this population has almost tripled since 1980. The Latino population is of considerable importance for public health and epidemiological research in the US because of its size and growth rate, and because this population includes a complex mix of people from different countries, of differing economic status, different preferred languages for various social, work, or media related activities, and varied level of acculturation to Non-Latino U.S. society.

A key challenge for measuring health behaviors and conducting epidemiological studies in the Latino population is the development of comparable survey instruments capable of eliciting valid and repeatable responses concerning both health behaviors and the psychological and social aspects of the recent immigrants [e.g., [1]]. Such efforts are important because valid measures of health behaviors are needed to address health disparities. This paper addresses the challenge of designing standardized survey questions for public health surveillance and research for populations containing significant numbers of immigrants, who tend to exhibit limited acculturation to U.S. society, at least initially [2]. We build on a recent analysis of acculturation and physical activity in Latino immigrants [3]. That investigation reported that self-reported leisure-time physical activity levels were greater in Latinos who had a greater degree of language acculturation. Such an association could arise because of a change in behavior associated with language acculturation. However, it could also have emerged due to between-group differences in the interpretation of questions related to physical activity frequency and intensity, absent any behavior change. The present study addresses reporting of survey items on acculturation, and on physical activity, to assess potential variation in interpretation between individuals of varying levels of residence time in the US, and of varied fluency in English.

Acculturation measures have been difficult to develop for health surveys, especially because these are the subject of considerable controversy, as an explanatory variable concerning health status and health behaviors. Some studies document strong correlations between health behaviors and acculturation, as measured by language use or duration of residence in the US [3-7]. In contrast, recent critiques of the use of acculturation as an explanatory variable for positive and negative changes in health behaviors in immigrant populations argue that use of language or residence time as proxies for acculturation obscures more complex and poorly defined cultural differences and their transformation over time [8]. Additional critiques have emphasized inadequate control for socioeconomic factors and the possibility that acculturation is a multidimensional process [1,8-11].

### Cross-cultural instrument pretesting

Population-based research and surveillance requires valid instruments for the assessment of lifestyle variables and for demographic and cultural variables that might influence behavior. Of particular concern is the possibility that language and cultural variation in immigrant populations might make it difficult to develop such instruments so as to produce equally valid results for people with different nativities and language skills - or cross-cultural comparability [11-13]. Cognitive interviews have emerged as a key tool for analyzing and improving standardized survey questions [14], and increasingly, researchers have utilized cognitive techniques to the development and improvement of questionnaires designed for use in multi-lingual or multi-cultural populations [14,15]. Development of survey items for particular populations may also require use of culturally specific examples; for physical activity, time use surveys could be a useful tool to identify common activities for population subgroups [16].

Cognitive interviewing involves the intensive study of how survey questions are interpreted, how information is recalled, and how respondents make decisions to provide a particular response [14]. Small groups of individuals are recruited for intensive interviews by specially-trained interviewers, who apply cognitive probes (e.g., "What does the term 'moderate physical activity' mean to you?"). On the basis of responses to such probes, the evaluated questions are modified to eradicate problems, especially those having a cognitive origin. Generally, problems identified in cross-cultural cognitive interviewing studies can involve either errors of translation, or more general design problems [17]. More general design problems have been divided into various categories. For example, the Q-BANK database of cognitive testing reports http://wwwn.cdc.gov/QBANK/Home.aspx, systematically characterize the types of problems observed in the cognitive pretesting of survey questions into nine specific coding categories: (1) Interviewer Difficulties, (2) Problematic Terms, (3) Ambiguous Concepts, (4) Overly Complex Question, (5) Erroneous Assumption or Double Barrel Question(6) Questionnaire Effects (spanning more than a single item), (7) Recall/Estimation Difficulty, (8) Biased/Sensitive Question, and (9) Inadequate Response Options. Q-BANK codes have been used as part of an interagency Federal effort to characterize the outcomes of cognitive interviews, and as a compendium of the types of flaws commonly exhibited by a range of evaluated survey questions. Similarly, Willis et al have divided such problems into six categories; interviewer difficulty, question wording, question structure, recall and judgment, response selection, and other problems. These include features producing difficulties related to cognitive processing (comprehension, information retrieval, decision-making, or response production) and problems of cultural adaptation such as the tendency to give open ended responses to some questions (a response selection problem) or generic cognitive problems with questions that occur regardless of language such as recalling the number of times a respondent felt something) [14,17-19].

Cognitive interviews for cross-cultural and multilingual studies are not a panacea, as challenges have been identified such as difficulties in understanding cognitive probe questions [20,21], variation in interviewer experience [22,23], and cultural barriers to honest articulation of opinions concerning question meaning [24]. Nevertheless, cognitive interviews do appear to be a useful tool for identifying language and cultural problems associated with questionnaires aimed at diverse populations [12,15,18,19,22,23,25-27].

### Cross-cultural variation due to question topic

Cross-cultural cognitive studies have investigated a variety of types and categories of survey questions. However, these have not explicitly and systematically sought to determine whether qualitatively different types of questions function in discrepant ways across cultural groups. That is, although several researchers have observed sources of cross-cultural non-equivalence with respect to individual survey questions [17,20,27-30], these studies have not generally sought to chronicle patterns, such that we can conclude that particular categories of questions pose particular challenges to cross-cultural administration and interpretation. Further, researchers have only begun to examine acculturation level and residence time, in addition to nominal socio-cultural group membership or language use, as influences on the survey response process.

Presumably, some topics or question areas will create particularly acute cross-cultural discrepancies. For example, several authors have suggested that questions relating to social systems that differ across countries or groups (health insurance systems, educational systems, foster-care arrangements, dietary meal patterns) will be likely to create cross-cultural challenges, given that the underlying assumptions on which the questions are based do not universally apply [28,31]. On the other hand, some behaviors that are general in nature, such as activities of everyday life, or physical activity, may be general enough that they share an underlying commonality, such that questions that are posed to one group should presumably apply to other. Therefore, for the current study, we focused on the conduct of cognitive interviews of Latino respondents with different residency times and use of English versus Spanish, to examine responses to two distinguishable categories: (a) questions about physical activity, and (b) those pertaining to acculturation to U.S. society.

### Cross-cultural aspects of physical activity items

It is not clear how questions on physical activity should be expected to function cross-culturally. Despite the fact that this topic is logically relevant across groups (as everyone engages in activity, or is inactive, to varying degrees), it has been well-established that certain types of items in this content domain in fact fail the fundamental test of cross-cultural equivalence, especially when they have originally been developed for White, Non-Latino respondents. For example, Ainsworth [32,33] has argued that in taking a detailed, checklist approach to assessing specific physical activities, questionnaire designers must take care to assure that they have included the appropriate list of items for all groups, rather than those that pertain mainly to a dominant or reference population. For instance, rather than asking only about activities (e.g., exercise classes) that are engaged in by a middle-class population, the questions must account for those behaviors that represent the physical activities of low-income Latino women.

Given the difficulty of "asking the right question" in this sense, an alternative potential approach is to pose questions that do not attempt to cover specific activities, but rather generalize the questions to a point where the key elements asked about should apply to all groups. So, in assessment of physical activity, rather than asking about particular behaviors, we chose to use more general questions from the 2000 National Health Interview Survey concerning light and moderate activity (see below). Given that this approach departs from that of identifying specific activities, it should no longer matter which component behaviors actually give rise to these physiological effects. Hence, we hypothesized that a series of physical activity questions that were stated in this general manner, and that covered behaviors which are presumably universal, such as walking, would minimize the influence of residence time and language choice. On the other hand, one could emphasize the fact that these items were designed mainly with the white, non-Latino population in mind, and the items could produce unforeseen problems, particularly for recent immigrants.

### Cross-cultural aspects of acculturation items

As a second topic which was intended to explicitly involve issues likely to interact with cultural background and language use, we chose to assess questions on self-reported acculturation level, designed for Latinos [10,34-38]. Such questions ask about language use, ethnic composition of peer groups, etc., and are therefore presumed to be applicable to all Latinos. One might expect these items to perform quite well across the full range of Latinos, especially as they have been specifically targeted to that particular group, and were not designed (as are many health-survey questions) for administration to a Non-Latino population.

Alternatively, an overt emphasis on cultural factors might itself produce complications when administered to Latino sub-groups of varying levels of residence time, to the extent that members of these sub-groups respond differentially in ways that induce measurement error. In other words, if cultural variables associated with respondent characteristics interact with those associated with question characteristics, then cross-cultural non-equivalence would seem to be the inevitable effect. By conducting cognitive interviews with Latinos of varying backgrounds, in English and Spanish, we endeavored to test the robustness of commonly used acculturation-related items, and to produce results that would support one or the other of the above hypotheses. Further, we attempted to determine whether observed problem in either instrument concerned difficulties with translation (that is, conversion of terms and whole questions from English to Spanish); versus more general types of questionnaire-design defects.

## Methods

### Questions

Physical activity questions were selected from a large, ongoing Federally-sponsored population health survey, the 2005 National Health Interview Survey http://www.cdc.gov/nchs/nhis/nhis_2005_data_release.htm. These questions had already been translated into Spanish and used extensively in the implementation of the 2005 NHIS. We used already-translated physical activity items because they were from one source (the National Health Interview Survey), and the procedures were known and deemed to have been effective [see, e.g., [39]]. Note also that these translations were further reviewed by the translators used for the acculturation related questions.

The selected questions categorized physical activities in terms of intensity and function; e.g., "How often do you do LIGHT OR MODERATE LEISURE-TIME physical activities for AT LEAST 10 MINUTES that cause ONLY LIGHT sweating or a SLIGHT to MODERATE increase in breathing or heart rate?" Questions also asked the respondents to estimate the duration of such activities; e.g. "About how long do you do these light or moderate leisure-time physical activities each time?" Similar questions concerning vigorous activity, leisure walking, and transportation walking were also included in the cognitive test (Additional file 1).

We hypothesized that a series of physical activity questions that were stated in this general manner and that covered behaviors such as walking, which are presumably universal, would serve to ameliorate differences between Latinos and Non-Latinos, and between English and Spanish language of administration. On the other hand, one could emphasize the fact that these items were designed mainly with the white, non-Latino population in mind, and the items could produce unforeseen problems for recent immigrants in particular.

Acculturation questions were selected based on a literature review of extant survey instruments. We did not attempt to directly address the debates over the dimensionality of acculturation or its role in society, but rather selected items from across the spectrum of instruments that represent these viewpoints. A review identified 21 distinct instruments designed to measure acculturation, and we selected questions from four surveys [ARSMA-Acculturation Scale for Mexican Americans [34-36]; GAI - General Acculturation Index [37]; LAECA -Los Angeles Epidemiologic Catchment Area Acculturation Scale; and AMAS - Abbreviated Multidimensional Acculturation Scale [10]]. These questions addressed (1) language use, (2) demographics, (3) friends and neighbors, (4) attitudes, and (5) behaviors. We tried to select items spanning as wide a range of phrasing and formatting as possible, based on review of all 21 instruments. Complete text of items in English and Spanish and their source are given in the supplementary materials.

Although some of the selected acculturation questions were already translated, we chose to re-translate them, because we were unsure of the procedures that had been used for previous instruments and we wanted to ensure consistent translation procedures, and quality, across all questions. The acculturation items were translated by a native Spanish speaker of Central American origin. The survey translation standards used by the U.S. Census Bureau [40] and the European Social Survey [41] recommend following the initial translation step with separate review and adjudication steps [22,23]. Increasingly, procedures that rely on careful, team-based forward translation are supplanting the sole use of back-translation as a favored practice for question translation [40,42,43]. Following these guidelines, two independent reviewers examined the newly translated acculturation questions as well as the previously translated physical activity questions.

Following this review, the adjudicator made relatively minor changes to the acculturation item translations before cognitive testing. Examples of the types of changes included slight wording changes (e.g., "cultura americana" to "cultura norteamericana estadounidense); grammatical corrections (e.g., "caminando" to "el caminar"); less formality in speech (e.g., "en general" to "por lo general"); use of more common language (e.g., changed "algo bien" ["somewhat well"] in a response category to "mas o menos bien", roughly equivalent to "more or less"); and a change in how frequency was asked (e.g., "Que tan seguido" ["how often"] was changed to "con que frequencia" ["how frequently"]). The adjudicator also made slight changes to the physical activity questions, consisting of remediating inconsistencies in the language used (e.g., "aumentan" consistently instead of "incrementan ") and editing wording felt to be awkward (e.g., "que usted acostumbra" ["that you usually "], was changed to "en relación" ["relating to," or "in relation to "]).

Full text of the physical activity and acculturation questions in English and Spanish and associated initial probes concerning responses to and interpretations of the questions are presented in Additional File 1.

### Recruitment

Three groups of nine respondents were recruited in the Washington DC suburbs and in Denver, Colorado). In each location, interview candidates were screened, recruited, and interviews were scheduled with volunteers who met study requirements. The study design explicitly included respondents from a range of Latino and Latino national backgrounds (Table 1). Respondents were either Spanish-speaking or bilingual in English and Spanish, were born somewhere other than the U.S., and represented a mix of ages and education levels. Respondents were recruited from a variety of sources, including a proprietary database of study volunteers, responses to fliers, and a local site in Maryland that serves a large Latino population. The volunteer respondents were assigned to one of the three design conditions based on whether they preferred to complete an interview in English or Spanish, and on the length of time they lived in the U.S. A total of 27 interviews were completed, nine each in the following three groups: (a) low residence time (< 5 years in the US)/Spanish, (b) l5+ years in U.S/Spanish, and (c) l5+ years in U.S/English.

**Table 1:** Study design and demographic characteristics of cognitive interview subjects

	Group 1: **Spanish interview**, **< 5 years in U.S**. (n = 9)	Group 2: **Spanish interview**, **l5+ years in U.S**. (n = 9)	Group 3: **English interview**, **15+ years in U.S**. (n = 9)
Location of Interviews	Maryland (MD)	Maryland/Colorado	Maryland/Colorado
Gender			
Male	5	4	4
Female	4	5	5
			
Latino Subgroup			
Caribbean	1	1	1
Central American	3	3	3
South American	2	2	2
Mexican	3	3	3
			
Level of Education			
0	1	0	0
1-6	3	0	0
7-8	1	0	0
9-12	2	7	4
College graduate	1	2	4
Technical School	1	0	2
			
Age			
18-29 yrs	2	1	1
30-39 yrs	1	3	2
40-49 yrs	4	3	3
50-59 yrs	1	1	2
60+ yrs	1	1	1
			
Interview Location			
Rockville, MD	9	6	7
Denver,CO	0	3	2

### Cognitive Interviews

Study procedures and materials were approved by Westat's Internal Review Board (IRB), and determined to be exempt from IRB review at NIH. Two trained bilingual cognitive interviewers (one of Mexican background and one Puerto Rican), conducted the 18 interviews for Spanish-language cognitive interviews. Three additional trained cognitive interviewers conducted the 9 interviews for English-language cognitive interviews. The lead interviewer for all three design conditions had additional qualitative research, survey design, questionnaire translation and cognitive interview analysis skills. The lead interviewer coordinated all interviewing and reporting activities and conducted roughly half of the cognitive interviews.

A senior staff member conducted a four-hour study specific training session that reviewed project goals and procedures. Building on Goerman's [21,44] guidelines, the training stressed the importance of administering draft survey questions as worded, while remaining flexible about administering cognitive interview probe questions. Some examples of standardized probes include the following, "Why did you say (answer)?" and "How easy or difficult was it for you to come up with an answer?" The training included several role-play exercises to practice cognitive interviewing skills. In addition, senior project staff monitored interviews and provided feedback to interviewers periodically throughout the data collection period.

Most (n = 14) of the interviews took place in cognitive laboratory facilities at Westat in Rockville, Maryland, and others took place at community day labor sites in Maryland (n = 10) and in Denver, Colorado (n = 3). Study subjects received $50 compensation for their participation. Interviews lasted approximately 60 minutes and followed a cognitive interview protocol with two main elements. First, interviewers explained the purpose of the project and the structure of the cognitive interview, and assured respondents that all information would be treated as confidential. Interviewers requested permission to audiotape interviews and, when applicable, informed respondents that Westat staff were observing the interview. Interviewers reminded respondents that they could refuse to answer any questions and that they could end the interview at any time; respondents provided written informed consent. The draft questionnaire was administered using concurrent cognitive probing, in which respondents answered the tested questions as the interviewer read them, and interviewers also administered structured probes that assessed how respondents interpreted key questions or phrases and how they selected questionnaire responses. Cognitive interviewers also used spontaneous probe questions (those that were unscripted) at any point in the interview to follow up any observed confusion or difficulties answering the questionnaire items [14].

Interviewers took minimal notes during the interview in order to concentrate on the conversation, and then reviewed the audio recordings of their interviews and prepared detailed summaries using a standard summary template designed by senior team members to ensure complete reporting.

### Analytic Approach

Table 2 gives an overview of the 4-step analytic process we used to examine these qualitative data Our data analysis approach reflects recommendations by Conrad and Blair [45] that pretest analyses should be structured first to interpret interview results to identify problems, and then to code identified problems by type or category.

**Table 2:** Overview of four-step qualitative analytic process

Step	Analytic methods	Analytic products	Goal
Step 1	Qualitative data reduction	Review audiotaped interviews and summarize key findings by item, separately for each cognitive interview respondent	Interpret interview results and document evidence of problems or issues in each interview
Step 2	Problem identification and classification	Identify problems: For each interview, identify items with evident problems or issues	Determine items with issues to be coded, separately for each pretest interview
		Classify general types of problems or issues observed for each item in each interview	Characterize general types of problems evident within individual interviews
Step 3	Item-level synthesis	Catalog problems and issues observed across interviews	Determine items with consistent problems or issues and document types of problems and issues observed consistently, by item
Step 4	Residence and topic area comparisons	Compare counts of problem items and problem types by residence time and question topic area	Assess whether types of problems observed differed depending on residence time or question topic

In analysis **Step 1: Narrative Summarization: **Cognitive interviewers reviewed the audio-taped interviews to identify and document key findings for each tested item in each cognitive interview. Based on these review activities, interviewers produced a narrative summary (in English) for each pretest interview.

Analysis **Step 2: Problem Identification**, involved reviewing interview summaries and identifying problems or issues. Members of the research team ('analysts') identified an item as having problems or issues when an interview summary contained evidence of response error or the potential for extraneous response variance due to difficulties understanding the question, or difficulties selecting a response. For example, in one question, respondents were asked, "In what language are the radio programs you usually listen to? ", and we found that respondents who did not listen to the radio were not able to answer this question. Another example illustrating this point involved the item, "What ethnic identification (does/did) your mother use?" Most respondents did not understand the term "ethnic identification" and were unable to answer the question correctly, or at all. At this point, analysts recorded whether a problem or issue was present was recorded.

Two analysts reviewed all 27 interview summaries. One analyst was not fluent in Spanish but had considerable experience with survey methods, questionnaire design, questionnaire translation for a range of languages (including Spanish) and questionnaire pre-testing. When this analyst disagreed with interviewer decisions, concerning whether the summary illustrated evidence of a problem or defect with the item, she added annotation on reasons for revising coding decisions and evidence used.

A second analyst then reviewed the interview summaries, the revised decisions and the accumulated documentation concerning presence or absence of problems in each interview. This second analyst was fluently bilingual in English and Spanish, and had experience with survey methods, questionnaire translation and cognitive interview testing. Analysis Step 2 produced a list of items along with descriptions of problems or issues.

In Analysis Step 3: **Coding**: Analysts reviewed the results and documentation from analysis Step 2, separately for each interviewed group. Analysts assigned a problem code only when there was evidence that two or more respondents in a design condition experienced the problem or issue.

In order to code, analysts relied on an abbreviated version of the Translation, Cultural Problem, Generic Problem (TCG) scheme introduced by Willis et al. [17]. The investigators regarded it critical to determine whether a problem was due to errors in translation, so that category was retained. Remaining problems were those that were due to problems other than translation, and that can in principle be distinguished as either (a) Generic problems of questionnaire design; or (b) Socio-cultural problems that are specific to a particular cultural group. However, given the current design, which involves members of one cultural group (Latinos), it was not possible to distinguish these, so these categories were combined into an overall category labeled Non-Translation errors. When assigning codes, the analysts summarized across all interviews within each of the three subject groups, to determine whether the most serious problem with each item was a Translation or Non-Translation issue; and to briefly summarize the overall finding for that item in qualitative, textual format. In order to more precisely characterize the nature of the observed problems, we also applied a further, more detailed coding system, adapted from the Q-BANK database of cognitive testing reports http://wwwn.cdc.gov/QBANK/Home.aspx. As introduced above, this system includes nine specific coding categories: (1) Interviewer Difficulties, (2) Problematic Terms, (3) Ambiguous Concepts, (4) Overly Complex Question, (5) Erroneous Assumption or Double Barrel Question(6) Questionnaire Effects (spanning more than a single item), (7) Recall/Estimation Difficulty, (8) Biased/Sensitive Question, and (9) Inadequate Response Options.

Analysis Step 4: **Compilation **involved computing counts of problems and types of problems separately by group (i.e., Spanish short residence time, Spanish longer residence time, and English longer residence time), and by question topic area (i.e., acculturation and physical activity). These counts provide the basis for assessing effects of residence time, language use and question topic area on question functioning.

### Tabulation, Presentation, and Statistical Analysis

We present a condensed tabulation of problems and a more inclusive tabulation. The condensed tabulation includes only problems identified in two or more respondents within a design condition; the more inclusive tabulation includes every problem. Table 3 illustrates the presence of translation or non-translation related problems, by design condition; and Table 4 depicts the results of the more detailed Q-BANK coding system. We report statistical tests using chi-squared statistics performed in SAS JMP Version 7.0 (SAS Institute, 2007, Cary, NC).

**Table 3:** Percent of tested items with translation and non-translation problems by question topic area and subject group.

	Percent of tested items (count) with one or more problems identified by two or more respondents		
	Translation	Non-Translation	Total
Design condition			
			
**Physical activity items**			
Spanish, < 5 years in US	0% (0)	69% (11)	11 (69%)
Spanish, 15+ years in US	6% (1)	62.5% (10)	11 (68.7%)
English, 15+ years in US	0% (0)	50% (8)	8 (50%)
			
**Average % with problems (across 16 items)**	**2%**	**62.5%**	
			
# Items tested = 16			
			
**Acculturation items**			
Spanish; < 5 years in US	4% (1)	93% (27)	28 (97%)
Spanish, 15+ years in US	4% (1)	62% (18)	19 (66.6%)
English, 15+ years in US	0% (0)	69% (20)	20 (69.0%)
			
**Average % with problems (across 29 items)**	**2%**	**75%**	
			
# Items tested = 29			

* See Table 4, here we summarize only problems where at least two respondents expressed concern over the same type of problem.

**Table 4:** Numbers of identified problems, by detailed problem type, design condition and questionnaire topic.^1^

	Acculturation Items^2^			Physical Activity Items^2^		
	Spanish; short time	Spanish; longer time	English; longer time	Spanish; short time	Spanish; longer time	English; longer time
1) Interviewer difficulty	8	6	6	1	0	0
2) Question wording	11	12	9	10	7	3
3) Question structure	1	0	0	0	0	0
4) Recall and judgment	0	0	0	1	4	5
5) Response selection	6	3	4	1	0	0
6) Translation	2	2	0	1	1	0
7) Other				0	0	0
						
TOTAL # problems	28	23	19	14	12	8

1 In this table we include all problems, even when only one respondent expressed a concern.

**2 Spanish; short time **= respondents who preferred to complete interviews in Spanish and who lived in the U.S. less than 5 years. **Spanish; longer time **= respondents who preferred to complete interviews in Spanish and who lived in the U.S. 15 years or more**. English; longer time **= respondents who preferred to complete interviews in English and who lived in the U.S. 15 years or more

## Results

The purpose of this study was to investigate the impact of residence time and language preference on the likelihood of experiencing problems with survey questions. Summarizing across question domain and problem type, the frequency of problems reported by two or more respondents ranged from 86% in low residence time/Spanish respondents to 67% and 62% respectively in high residence time/Spanish and English speaking groups. Translation problems were found to be infrequent across residence time/language conditions and question topic areas, suggesting that translation processes were effective (Table 3). On the other hand, a variety of non-translation problems were identified, for all three subject groups, and for both question topic areas (Table 3 and below).

Three main results are relevant. First, for both physical activity and acculturation items, the percentage of items exhibiting problems was marginally higher for acculturation-related questions (Chi^2^, p = 0.0524). Second the number of problems differed significantly by language group (Chi^2^, p = 0.0204), with the fewest problems for English interviews related to physical activity (50% of questions) and the most for low acculturated people interviewed in Spanish (97% of questions). These p values have to be interpreted with caution, as some observed and expected cell sizes are below five.

### Acculturation Items: Key Qualitative Findings

Given our small sample sizes, and lack of control for potentially confounding effects, we relied on intensive qualitative analysis of observed problems to identify potential sources and causes. For acculturation items, non-translation problems were especially prevalent for the less-acculturated subjects. A particular item format seemed to cause many difficulties, as illustrated below. For these items, field interviewers use a pre-coded set of categories to code open-ended responses (that is, the question is asked open-ended, but then coded into a pre-selected set of response categories).

Where was your mother born? (RESPONDENTS REPORT AN OPEN-ENDED ANSWER AND INTERVIEWERS CIRCLE A NUMBER TO CODE THE RESPONSE).

UNITED STATES...................................................1

MEXICO...............................................................2

CUBA...................................................................3

PUERTO RICO......................................................4

DOMINICAN REPUBLIC.........................................5

CENTRAL AMERICA (Name of Country).................6

         SOUTH AMERICA (Name of Country)...........7

         Other..........................................................8

         DK...............................................................9

Six of the 29 acculturation items used this general format, which produced difficulties for interviewers because respondents often reported the names of villages, towns or regions. If interviewers were unfamiliar with the countries respondents meant to refer to, then additional unscripted probing was necessary to identify the appropriate country or territory. Hence, this finding represents a classic response-matching problem [46], as subjects both understand the question and know the answer, but simply provide an answer at a different (geographic) level.

In reviewing these issues by group, we found that there was a decrease in problem frequency depending on how long the participant had been in the U.S., and a further decrease for those who also preferred to be interviewed in English. For those with less than 5 years in U.S., all nine respondents answered with the name of their village or town. Among those who were interviewed in Spanish but had been in the U.S. at least 15 years, 6 respondents answered with the name of the country (as intended), and 3 with the town or state. On the other hand, all nine of those who were interviewed in English and had been in the U.S. for at least 15 years answered with the name of the country.

Qualitative information revealed a further example of how interpretation of acculturation-related items may vary depending on how long the respondent has been in the U.S., and language preference. For the items: "When you were growing up, how many of your friends were of Anglo origin?" and "How many of your friends now are of Anglo origin?", most respondents who lived in the U.S. less than 5 years were found to be unfamiliar with the term "Anglo." Further, some respondents who lived in the U.S. 15 years or more and who preferred to complete the interview in Spanish were also unfamiliar with the term 'Anglo' and provided ambiguous explanations when asked to define it.

In contrast, all nine respondents who preferred to complete the interview in English were familiar with "Anglo." However, they did interpret the term in somewhat different ways in response to cognitive interview probes. Examples include "Anglo" as "from another nationality" (e.g., "non-Salvadoran; non-Puerto Rican"), "those who speak English", "non-Latino", "white" or "Caucasian"; "born in the U.S."; "white American"; "North American"; "of English descent"; and "northern European" (e.g., France, Germany, Holland, Switzerland). Because of the multiple interpretations of the term, we recommended that "Anglo" be replaced with an alternative, well-defined term that makes intended measurement goals clearer to all groups. "English Speaking European American" is a long but specific alternative. We are unaware of efforts to test this or other alternative phrases.

A further example of problems with acculturation-related items and involved the key term "ethnic identification" within the item *"What ethnic identification (does/did) your mother use?" *In general, the term "ethnic identification" was unfamiliar to respondents in all three design conditions. Several subjects who lived in the U.S. less than 5 years adopted an unexpected interpretation of the item, inferring that "ethnic identification" referred to official paperwork related to proof of citizenship or legal status (e.g., "your certificate of baptism" or "birth certificate"). This seems important, because absent cognitive testing, it is not obvious that this problem would be detected from respondent responses and the resulting misclassification would go undetected.

Further, testing of the follow-up question *"Would you say she is/was Latino, Hispanic, American, North American, Cuban, Mexicano, or something else? "*, which was asked when respondents were unable to reply in open-ended form, yielded further evidence of interpretive variation across groups. Most respondents who had lived in the U.S. less than five years were unfamiliar with terms such as "Latino" or "Hispanic ". However, several subjects in the two groups who lived in the U.S. 15 years or more also failed to identify with any of the response options, stating that these labels over-simplify ethnic background by glossing over important distinctions among groups from different regions and groups with different nationalities. This example suggests that items on ethnic background are problematic, but may present somewhat different problems for more and less acculturated respondents.

Respondents also had trouble with categories for language and thinking activities, such as with the categories "only Spanish", "mostly Spanish", "Spanish and English about the same," "mostly English", "only English", or "another language ". The qualifiers 'only' and 'mostly' were either ignored or did not fit respondents' situation. The phrase "In which language do you think?" was sometimes interpreted by respondents to mean, "What language do you think about. "

### Physical Activity Items: Key Qualitative Findings

For the physical activity questions, question design issues (as opposed to translation problems) were again common, but appeared to affect all subject groups equally, from both quantitative and qualitative perspectives. Problems mainly related to either vague wording, difficulties in recalling necessary information, or estimating a response. Vague terms that respondents in all three groups identified within the physical activity items included "vigorous activity", "light or moderate" activities, "leisure" activities and "physical activities specifically designed to strengthen your muscles." Respondents had problems consistently recalling information and estimating activity frequency and activity duration across the set of items on walking and exercise activities. For example, in response to probe questions concerning walking for transportation, one respondent reported walking to the mall every day during the past 7 days. She stated that she knew that these walks were more than 10 minutes because she usually spent about 3 hours walking to, from, and around in the mall each time. On the other hand, for another question about walking, the same respondent reported walking about 15 minutes a day during the past 7 days. In general, question about vigorous activity involving lengthy definitions (e.g., activity for 10 minutes, that causes heavy sweating or large increases in breathing or heart rate...) had to be repeated multiple times due to question wording complexity.

Another subject answered "yes" to a question concerning walking, reporting that the prior day she had walked to a store (a walk that took her 20-25 minutes, round trip), and she also walked to get to the bus (a 12 minute walk). However, her responses to related questions suggested that it is challenging to summate these different episodes in a consistent manner. Finally, subjects in all three groups often provided answers in terms of ranges (e.g., "10 to 15 minutes") or multiple answers, that is, separate responses for different walking activities. These response patterns are an additional indication that respondents may have difficulty estimating the requested time duration. Again, these problems were fairly equally distributed across Latino subgroup tested.

Although cross-cultural differences were not strongly reflected in PA items, we did find some hints of cross-cultural variation. Concepts such as weekends or weekdays were ambiguous to some respondents. Further, some respondents did not divide their week into 5 weekdays and 2 weekend days. When asked: (a) *Outside of work, how many hours do you spend per day during **WEEKDAYS **sitting?; *and (b) *Outside of work, how many hours do you spend per day during the **WEEKEND **sitting*, some subjects who preferred Spanish for the interview reported that they were thinking of "either everyday, 5-6 working days, or that day" when asked about the specific term "weekday"; and that weekend included only Sunday, or else included Friday, Saturday, and Sunday. English speakers, on the other hand seemed to accept without comment that the weekend was Saturday and Sunday.

In one case, a translation problem arose where Spanish translation included words that conveyed the wrong meaning."Aficiones" was the word used to convey hobby even though in many Spanish-speaking countries it refers to a passion toward something. This kind of problem also arose for other words, but survey questions in other domains, beyond acculturation and physical activity, could well present even more translation problems due to meaning and variation in pronunciation.

### Specific Problems

There were differences in the specific kinds of problems reported for acculturation versus physical activity items that are apparent when problem types are categorized according to the Q-bank criteria. Table 4 presents the tabulation of problem types in the acculturation and PA domains for each of the interview conditions. PA questions resulted in more problems involving recall and judgment processes, whereas acculturation questions resulted in many more problems concerning interviewer difficulty and response selection. There were many problems related to question wording in both domains. This difference in the frequency of problem types across domains was statistically significant based on a chi-squared test comparing the frequency of problems between acculturation items and physical activity items (p < 0.05). Inspection indicates no evidence that language use was related to problem type in this more inclusive tabulation.

## Discussion

This study identified very few translation problems regardless of survey question topic and no evidence that such problems were more common for more recent immigrants or immigrants who preferred to be interviewed in Spanish. Both PA- and acculturation-related questions elicited non-translation related problems including problems largely related to interviewer difficulty, question wording and response selection. For the most part, it appears that despite the best efforts of designers survey questions appear to simply be difficult to interpret in exactly the manner intended by the designers. There was clear evidence that the kind of problem reported was related to question area and somewhat weaker evidence that residence time/language use was associated with more difficulties concerning acculturation related questions. Questions about PA vs. acculturation elicited distinct problem types; response selection and interviewer difficulty problems were common for acculturation related questions, whereas question wording problems and recall and judgment issues were most common for PA questions. We conclude from these observations that work to improve general question design characteristics could produce benefits across cultures and immigrant groups with different residence times. Such work should be informed by the fact that serious problems of multiple types can arise in standardized survey questions that go undetected in the absence of formal cognitive testing and that these problems can vary by question content.

A single study does not guarantee identification of all problems in a class of questions. For example, Altschuler et al (2009) describe a suite of problems identified in a PA-related questions from the *Life After Cancer Epidemiology Study *and *the California Men's Health Study *that differ considerably from those presented here, despite many apparent similarities between the survey items [47]. The cognitive approach can be used in the development of new physical activity instruments or the modification of existing instruments [48,49]. Durante and Ainsworth emphasize an approach based on the four basic stages of answering standardized survey questions; comprehension, retrieval, decision making, and response generation [48]. This framework appears to lend itself well to questions concerning duration and quantity such as those involving PA or diet. It is not as obvious how these stages map onto cultural variables or values and it is not apparent how they might differ in respondents of different cultural backgrounds. Cognitive testing of PA related questions has also indicated that occupational activity and heavy or vigorous activity are easier to recall than light or intermittent activity [49]. Interviewers trained in the use of calendars and memory probing improved the long term recall of PA, hinting at approaches that could be used in standardized survey questions such as calendar based prompts.

A second major point of this paper is to serve as a case study on the use of cognitive interviewing for comparisons of standardized survey item functioning among respondents using different languages and differing in relationship to other cultural and demographic variables. This approach could be invaluable in transnational surveillance efforts [e.g., [50-52]], and particularly in evaluating potential bias in international cohort studies, such as the EPIC study in Europe [53]. Such cohort studies often rely on the comparability of survey items to allow data pooling across countries and cognitive testing is a relatively economical tool to establish whether such comparisons are warranted. If problems are found to be equally well distributed across groups, error will at least be balanced. If, on the other hand, functioning of an item is found to systematically vary between groups, comparisons will introduce systematic bias that precludes meaningful cross-cultural comparison.

### Practical implications for question design

In one sense, it is fortunate that many of the problems observed were generic in nature, and occurred in multiple groups. At the least, this effect may serve to set a 'level playing field' in which cross-cultural differences are unbiased; although, variation in item interpretation within each group may add overall bias or variability to the set of obtained responses. To revisit one of our initial motivations, this would also suggest that changes in self-reported physical activity associated with changes in self reported acculturation are not likely to be caused by changing interpretation of physical activity questions. Nevertheless, the general U.S. population has difficulty with descriptors of physical activity such as 'intensity' [47] and there is no reason to believe that that would be any different for Latinos. There could also be some benefit to using examples of specific activities relevant to a given population. Focus groups could be useful to identify such activities.

More optimistically, increased attention to the basic principles of question design [e.g., [54]] could be effective in ameliorating problems across multiple language and cultural groups. Specifically, (a) attention to design characteristics could have benefit across groups, reducing the need for elaborate cross-cultural studies and (b) when cross-cultural pretesting is conducted, many of the results could reflect back to the source language version and lead to alteration of that version (a phenomenon sometimes referred to as "decentering"). This practice departs markedly from that of assuming that a source version (usually English) can be used as a set reference point and that translations should be developed to the point that they effectively mimic that version.

#### Caveats

This study has several major limitations. **First**, there were only 27 subjects divided across three groups. However, past work on cognitive testing and focus groups suggests that many of the dominant issues with standardized survey questions or topics addressed in focus groups emerge in modest sized groups [27,55]. Further, in cases where sample size is limited, qualitative researchers invoke procedures that "drill down" intensively into problem sources. To the degree that a coherent picture emerges pointing to a systematic explanation, it may be unnecessary to conduct sufficient testing for key explanatory variables to emerge statistically. Thus, we believe our sample size to be of sufficient numbers to demonstrate the presence of major types of problems with the survey items examined here. This, however, is admittedly an unresolved issue concerning the fundamental discrepancy in approaches between qualitative and quantitative research. **Second**, and related, is the fact that we cannot compare the responses of different Latino subgroups to questions in the two domains. Cultural differences associated with country or region of origin could influence cognitive responses to survey items. **Third**, education and residence time/language preference are confounded in this sample; the low residence time/Spanish group has much lower levels of education than the other two groups. It would be interesting to examine cognitive responses of well-educated recent immigrants to these questions. However, this may be a smaller and more difficult to recruit population. Additionally, short term residents who preferred to receive interviews in English are missing from this study. Linguistic isolation can influence participation in health surveys [56]. **Fourth**, this study sampled residents from two geographic locales, lacking respondents from areas that could be culturally distinct (e.g. the US-Mexican border, Florida, and others).

## Conclusions

Our focus is on the US Latino population, but lessons learned from this work are likely applicable to many countries and cultural groups [57,58]. Overall, this study highlights the importance of considering the ability of respondents from all cultural and residence time groups to understand and answer survey questions. General cognitive challenges predominate in questions concerning physical activity and other typical health behaviors. However, here we found that questions about acculturation status may result in particular difficulties for more recent immigrants who prefer to respond to questions in Spanish. Because of the growing immigrant population in the U.S. and global increases in migration, it is a public health priority to continue efforts to improve such questions for effective use in diverse populations.

In summary, the results of this study further complement recent work on the challenges associated with question design in the use of dietary questions in national and regional surveys [14,22,23]. Together these investigations suggest that questions about health behaviors, notably diet and physical activity, can be effectively developed for people from diverse cultural and linguistic backgrounds with comparable cognitive testing. Such problems appear to differ by topic area (i.e., for acculturation vs physical activity), but these differences do not appear to preclude the application of general survey question design rules to reduce error across different cultural groups defined by language use.

## Competing interests

The authors declare that they have no competing interests.

## Authors' contributions

DB and GW conceived of the project, analyzed data and drafted the paper. BF, CH, KL and AN, collected data, extracted and analyzed data, reviewed and rewrote sections of the paper. All authors read and approved the final manuscript.

## Pre-publication history

The pre-publication history for this paper can be accessed here:

http://www.biomedcentral.com/1471-2458/10/481/prepub

## Supplementary Material

Additional file 1 **Acculturation and physical activity questions and initial probes**. This file contains the questions examined via cognitive testing and the initial probes used in this study in both English and Spanish language versions. Click here for file

## References

[B1] Lara M Gamboa C Kahramanian MI Morales LS Bautista DE Acculturation and Latino health in the United States: a review of the literature and its sociopolitical context Annu Rev Public Health 2005 26 367 397 10.1146/annurev.publhealth.26.021304.144615 PMC5920562 15760294

[B2] Borjas GJ Assimilation and changes in cohort quality revisited: what happened to immigrant earnings in the 1980s? J Labor Econ 1995 13 201 245 10.1086/298373 12291239

[B3] Berrigan D Dodd K Troiano RP Reeve BB Ballard-Barbash R Physical activity and acculturation among adult Hispanics in the United States Res Q Exerc Sport 2006 77 147 157 10.1080/02701367.2006.10599349 16898271

[B4] Aldrich L Variyam JN Acculturation erodes the diet quality of U.S. Hispanics Food Rev 2000 23 51 55

[B5] Crespo CJ Smit E Carter-Pokras O Andersen R Acculturation and leisure-time physical inactivity in Mexican American adults: results from NHANES III, 1988-1994 Am J Public Health 2001 91 1254 1257 10.2105/ajph.91.8.1254 PMC1446756 11499114

[B6] Morales LS Lara M Kington RS Valdez RO Escarce JJ Socioeconomic, cultural, and behavioral factors affecting Hispanic health outcomes J Health Care Poor Underserved 2002 13 477 503 10.1177/104920802237532 PMC1781361 12407964

[B7] Neuhouser ML Thompson B Coronado GD Solomon CC Higher fat intake and lower fruit and vegetables intakes are associated with greater acculturation among Mexicans living in Washington State J Am Diet Assoc 2004 104 51 57 10.1016/j.jada.2003.10.015 14702584

[B8] Hunt LM Schneider S Comer B Should "acculturation" be a variable in health research? A critical review of research on US Hispanics Soc Sci Med 2004 59 973 986 10.1016/j.socscimed.2003.12.009 15186898

[B9] Ryder AG Alden LE Paulhus DL Is acculturation unidimensional or bidimensional? A head-to-head comparison in the prediction of personality, self-identity, and adjustment J Pers Soc Psychol 2000 79 49 65 10.1037//0022-3514.79.1.49 10909877

[B10] Salant T Lauderdale DS Measuring culture: a critical review of acculturation and health in Asian immigrant populations Soc Sci Med 2003 57 71 90 10.1016/s0277-9536(02)00300-3 12753817

[B11] Marin G Marin BV Bickman L, Rog DJ Research with Hispanic populations 1991 Newbury Park, CA: Sage Publications

[B12] Warnecke RB Johnson TP Chavez N Sudman S O'Rourke DP Lacey L Horm J Improving question wording in surveys of culturally diverse populations Ann Epidemiol 1997 7 334 342 10.1016/s1047-2797(97)00030-6 9250628

[B13] Ponce NA Lavarreda SA Yen W Brown ER DiSogra C Satter DE The California Health Interview Survey 2001: translation of a major survey for California's multiethnic population Public Health Rep 2004 119 388 395 10.1016/j.phr.2004.05.002 PMC1497648 15219795

[B14] Willis GB Cognitive interviewing: a tool for improving questionnaire design 2005 Thousand Oaks, CA: Sage Publications

[B15] Agans RP Deeb-Sossa N Kalsbeek WD Mexican immigrants and the use of cognitive assessment techniques in questionnaire development Hisp J Behav Sci 2006 28 209 230

[B16] Dunton GF Berrigan D Ballard-Barbash R Graubard BI Atienza AA Environmental influences on exercise intensity and duration in a U.S. time use study Med Sci Sports Exerc 2009 41 1698 1705 10.1249/MSS.0b013e3181a06c9b 19657302

[B17] Willis G Lawrence D Hartman A Stapleton KM Levin K Forsyth B Translation of a tobacco survey into Spanish and Asian languages: the Tobacco Use Supplement to the Current Population Survey Nicotine Tob Res 2008 10 1075 1084 10.1080/14622200802087572 PMC3803133 18584471

[B18] Willis GB Lawrence D Kudela M Levin K Miller K The use of cognitive interviewing to study cultural variation in survey response 2005 Paper presented to the Questionnaire Evaluation Standards (QUEST) Workshop on Questionnaire Design and Testing

[B19] Willis G Lawrence D Thompson F Kudela M Levin K Miller K The use of cognitive interviewing to evaluate translated survey questions: lessons learned Proceedings of the Federal Committee on Statistical Methodology Research Conference; Arlington, VA 2005

[B20] Pasick RJ Stewart SL Bird JA D'Onofrio CN Quality of data in multiethnic health surveys Public Health Rep 2001 116 223 243 10.1093/phr/116.S1.223 PMC1913670 11889288

[B21] Goerman P Adapting cognitive interview techniques for use in pretesting Spanish language survey instruments 2006 Washington, DC: Statistical Research Division, U.S. Census Bureau

[B22] Levin K Willis GB Forsyth BH Norberg A Kudela MS Stark D Thompson FE Using cognitive interviews to evaluate the Spanish-language translation of a dietary questionnaire Surv Res Methods 2009 3 13 25

[B23] Forsyth BH Kudela MS Levin K Lawrence D Willis GB Methods for translating an English-language survey questionnaire on tobacco use into Mandarin, Cantonese, Korean and Vietnamese Field Methods 2007 19 264 283

[B24] Pan Y The role of sociolinguistics in the development and conduct of federal surveys Proceedings of the Federal Committee on Statistical Methodology Research Conference 2003 Arlington, VA

[B25] Kudela MS Forsyth BH Levin K Lawrence D Willis G Cognitive interviewing versus behavior coding 2006 Paper presented to the American Association for Public Opinion Research, Montreal, Canada

[B26] Thompson FE Subar AF Brown CC Smith AF Sharbaugh CO Jobe JB Mittl B Gibson JT Ziegler RG Cognitive research enhances accuracy of food frequency questionnaire reports: results of an experimental validation study J Am Diet Assoc 2002 102 212 225 10.1016/s0002-8223(02)90050-7 11846115

[B27] Willis G Zahnd E Questionnaire design from a cross-cultural perspective: an empirical investigation of Koreans and non-Koreans J Health Care Poor Underserved 2007 18 197 217 10.1353/hpu.2007.0118 18065860

[B28] Napoles-Springer AM Stewart AL Overview of qualitative methods in research with diverse populations. Making research reflect the population Med Care 2006 44 S5 S9 10.1097/01.mlr.0000245252.14302.f4 17060835

[B29] Napoles-Springer AM Santoyo-Olsson J O'Brien H Stewart AL Using cognitive interviews to develop surveys in diverse populations Med Care 2006 44 S21 S30 10.1097/01.mlr.0000245425.65905.1d 17060830

[B30] Quittner AL Espelage DL Levers-Landis CE Drotar D Measuring adherence to medical treatments in childhood chronic illness: considering multiple methods and sources of information J Clin Psychol Med Settings 2000 7 41 54

[B31] Krause N The use of qualitative methods to improve quantitative measures of health-related constructs Med Care 2006 44 S34 S38 10.1097/01.mlr.0000245429.98384.23 17060833

[B32] Ainsworth BE Challenges in measuring physical activity in women Exerc Sport Sci Rev 2000 28 93 96 10902093

[B33] Ainsworth BE Issues in the assessment of physical activity in women Res Q Exerc Sport 2000 71 S37 S42 10925823

[B34] Cuellar I Harris LC Jasso R An acculturation rating scale for Mexican American normal and clinical populations Hisp J Behav Sci 1980 2 199 217

[B35] Cuellar I Arnold B Maldonado R Acculturation rating scale for Mexican Americans-II: a revision of the original ARSMA scale Hisp J Behav Sci 1995 17 275 304

[B36] Buki LP Borrayo EA Feigal BM Carrillo IY Are all Latinas the same? Perceived breast cancer screening barriers and facilitative conditions Psychol Women Q 2004 28 400 411

[B37] Castro FG Gutierres S Drug and alcohol use among rural Mexican-Americans NIDA Res Monogr 1997 168 498 530 9260179

[B38] Burnam MA Telles CA Karno M Hough RL Escobar JI Measurement of acculturation in a community population of Mexican Americans Hisp J Behav Sci 1987 9 105 130

[B39] Vadaparampil ST Wideroff L Breen N Trapido E The impact of acculturation on awareness of genetic testing for increased cancer risk among Hispanics in the year 2000 National Health Interview Survey Cancer Epidemiol Biomarkers Prev 2006 15 618 623 10.1158/1055-9965.EPI-05-0378 16614100

[B40] U.S. Census Bureau Census Bureau Guideline: language translation of data collection instruments and supporting materials 2004 Washington, DC: U.S. Department of Commerce

[B41] European Social Survey European Social Survey (ESS): translation strategy http://www.europeansocialsurvey.org/index.php?option=com_content&task=view&id=66&Itemid=112

[B42] Harkness JA Schoua-Glusberg A Harkness JA Questionnaires in translation Cross-cultural survey equivalence. ZUMA-Nachrichten Spezial Number 3 1998 Mannheim: ZUMA 87 128

[B43] Harkness JA van de Vijver FJ Mohler PP Cross-cultural survey methods 2003 Hoboken, NJ: Wiley

[B44] Goerman P Cognitive interview methodology revisited: development of best practices for pretesting Spanish survey instruments 2006 Paper presented to the American Association for Public Opinion Research, Montreal, Canada

[B45] Conrad FG Blair J Presser S, Rothgeb JM, Couper MP, Lessler JT, Martin E, Martin J, Singer E Data quality in cognitive interviews: the case of verbal reports Methods for testing and evaluating survey questionnaires 2004 New York: John Wiley & Sons, Inc. 67 89

[B46] Tourangeau R Jabine TB, Straf ML, Tanur JM, Tourangeau R Cognitive sciences and survey methods Cognitive aspects of survey methodology: building a bridge between disciplines 1984 Washington, DC: National Academy Press 73 100

[B47] Altschuler A Picchi T Nelson M Rogers JD Hart J Sternfeld B Physical activity questionnaire comprehension: lessons from cognitive interviews Med Sci Sports Exerc 2009 41 336 343 10.1249/MSS.0b013e318186b1b1 PMC2694667 19127192

[B48] Durante R Ainsworth BE The recall of physical activity: using a cognitive model of the question-answering process Med Sci Sports Exerc 1996 28 1282 1291 10.1097/00005768-199610000-00012 8897386

[B49] Friedenreich CM Courneya KS Bryant HE The lifetime total physical activity questionnaire: development and reliability Med Sci Sports Exerc 1998 30 266 274 10.1097/00005768-199802000-00015 9502356

[B50] Craig CL Marshall AL Sjostrom M Bauman AE Booth ML Ainsworth BE Pratt M Ekelund U Yngve A Sallis JF Oja P International physical activity questionnaire: 12-country reliability and validity Med Sci Sports Exerc 2003 35 1381 1395 10.1249/01.MSS.0000078924.61453.FB 12900694

[B51] Abu-Omar K Rutten A Relation of leisure time, occupational, domestic, and commuting physical activity to health indicators in Europe Prev Med 2008 47 319 323 10.1016/j.ypmed.2008.03.012 18455785

[B52] Guthold R Ono T Strong KL Chatterji S Morabia A Worldwide variability in physical inactivity a 51-country survey Am J Prev Med 2008 34 486 494 10.1016/j.amepre.2008.02.013 18471584

[B53] Haftenberger M Schuit AJ Tormo MJ Boeing H Wareham N Bueno-de-Mesquita HB Kumle M Hjartaker A Chirlaque MD Ardanaz E Andren C Lindahl B Peeters PH Allen NE Overvad K Tjonneland A Clavel-Chapelon F Linseisen J Bergmann MM Trichopoulou A Lagiou P Salvini S Panico S Riboli E Ferrari P Slimani N Physical activity of subjects aged 50-64 years involved in the European Prospective Investigation into Cancer and Nutrition (EPIC) Public Health Nutr 2002 5 1163 1176 10.1079/PHN2002397 12639225

[B54] Willis GB Lessler LT The question appraisal system: a guide for systematically evaluating survey question wording 1999 Final report submitted to the Centers for Disease Control and Prevention, National Center for Chronic Disease Prevention and Health Promotion. Research Triangle Institute

[B55] Morgan DL Focus groups as qualitative research 1997 London: Sage

[B56] Link MW Mokdad AH Stackhouse HF Flowers NT Race, ethnicity, and linguistic isolation as determinants of participation in public health surveillance surveys Prev Chronic Dis 2006 3 A09 PMC1500943 16356362

[B57] Hosper K Klazinga NS Stronks K Acculturation does not necessarily lead to increased physical activity during leisure time: a cross-sectional study among Turkish young people in the Netherlands BMC Public Health 2007 7 230 10.1186/1471-2458-7-230 PMC2034552 17767715

[B58] Hosper K Nierkens V van Valkengoed I Stronks K Motivational factors mediating the association between acculturation and participation in sport among young Turkish and Moroccan women in the Netherlands Prev Med 2008 47 95 100 10.1016/j.ypmed.2008.02.024 18378289

