# Supplementary material for: Cognitive testing of physical activity and acculturation questions in recent and long-term Latino immigrants
Source: BMC Public Health. 2010 Aug 13;10:481. doi: 10.1186/1471-2458-10-481 (PMC2927546; doi:10.1186/1471-2458-10-481)
Supplement: Additional file 1 — Acculturation and physical activity questions and initial probes. This file contains the questions examined via cognitive testing and the initial probes used in this study in both English and Spanish language versions. [file 1471-2458-10-481-S1.DOC]

ACCULTURATION and PHYSICAL ACTIVITY QUESTIONS and Initial Probes

References for question origins are given in the methods section of the paper.

**Acculturation Questions**

Introduction: We are going to be asking you questions today about two main areas—acculturation and physical activity. By acculturation, we mean the degree to which you have adopted the American or Anglo culture. The physical activity questions will focus on things like exercise and walking. Okay, let’s get started.

Source: NHIS, 2000

**I. Language**

1. In general, which language do you *speak?* Would you say……(READ CATEGORIES AND CIRCLE NUMBER FOR R's ANSWER)

Only Spanish, 1

Mostly Spanish, 2

Spanish and English about the same, 3

Mostly English, 4

Only English, or 5

Another language? 6 Specify_____________

REFUSED 7

DK 8

- - **How did you decide on your answer? (How did you decide that you speak ________ the most?)**
  - **Did the choices offered to you fit your situation? If not, please say more about that.**

2. Which language did you use as a child? Would you say….

Only Spanish, 1

Mostly Spanish, 2

Spanish and English about the same, 3

Mostly English, 4

Only English, or 5

Another language? 6 Specify_____________

Refused 7

DK 8

- - **How did you decide on your answer? (How did you decide that you spoke ________ the most?)**
  - **What did you consider “a child” to mean?**

3. In general, which language do you *read* better? Would you say….

Only Spanish, 1

Spanish better than English, 2

Spanish and English about the same, 3

English better than Spanish, or 4

Only English? 5

Don’t read 6

Refused 7

DK 9

- - **How did you decide on your answer? (How did you decide that you read ________ better?)**
  - **What types of reading materials were you thinking about?**

1. Which language do you usually speak at home? Would you say…..?

Only Spanish, 1

More Spanish than English, 2

Spanish and English about the same, 3

More English than Spanish, or 4

Only English? 5

REFUSED 7

DK 9

- - **How did you decide on your answer? (How did you decide that you speak ________ the most?)**

1. Which language do you usually speak with your friends? Would you say….

Only Spanish, 1

More Spanish than English, 2

Spanish and English about the same, 3

More English than Spanish, or 4

Only English? 5

Refused 7

DK 9

- - **How did you decide on your answer? (How did you decide that you speak ________ the most?)**

6. In which language do you usually think? Would you say….

Only Spanish, 1

More Spanish than English, 2

Spanish and English about the same, 3

More English than Spanish, or 4

Only English? 5

Refused 7

DK 9

- - **How did you decide on your answer? (How did you decide that you think in ________ the most?)**

7. In which language are the T.V. programs you usually watch? Would you say….

Only Spanish, 1

More Spanish than English, 2

Spanish and English about the same, 3

More English than Spanish, or 4

Only English? 5

Refused 7

DK 8

- - **How did you decide on your answer? (How did you decide that you watch ________ the most?)**
  - **What types of TV shows do you watch?**

8. In which language are the radio programs you usually listen to? Would you say …

Only Spanish, 1

More Spanish than English, 2

Spanish and English about the same, 3

More English than Spanish, or 4

Only English? 5

Refused 7

DK 8

- - **We just asked you several questions about language. How easy or difficult was it to answer these questions about language?**

**II. Information About Your Parents, Grandparents, and You**

9.a Where was your mother born? (Respondents will report an answer, if they can, and the interviewer will then circle the NUMBER FOR THE response).

United States 1

Mexico 2

Cuba 3

Puerto Rico 4

***(protocol should follow up***

***on which country)***

Central America (Name of Country) 5

South America (Name of Country) 6

Other 7

DK 9

9.b Where was your father born?

United States 1

Mexico 2

Cuba 3

Puerto Rico 4

***(protocol should follow up***

***on which country)***

Central America (Name of Country) 5

South America (Name of Country) 6

Other 7

DK 9

9.c Where was your mother’s father born?

United States 1

Mexico 2

Cuba 3

Puerto Rico 4

***(protocol should follow up***

***on which country)***

Central America (Name of Country) 5

South America (Name of Country) 6

Other 7

DK 8

- - Who **do you think we are asking you about in your family**?

9.d Where was your father’s father born?

United States 1

Mexico 2

Cuba 3

Puerto Rico 4

***(protocol should follow up***

***on which country)***

Central America (Name of Country) 5 _____________

South America (Name of Country) 6 _____________

Other 7

DK 8

- - Who **do you think we are asking you about in your family**?

9.e Where was your mother’s mother born?

United States 1

Mexico 2

Cuba 3

Puerto Rico 4

***(protocol should follow up***

***on which country)***

Central America (Name of Country) 5 ______________

South America (Name of Country) 6 ______________

Other 7

DK 8

9.f Where was your father’s mother born?

United States 1

Mexico 2

Cuba 3

Puerto Rico 4

***(protocol should follow up***

***on which country)***

Central America (Name of Country) 5 ______________

South America (Name of Country) 6 ______________

Other 7

DK 8

No sabe……….………………………………8

- - **How easy or difficult was it for you to answer these questions about your grandparents?**

Source: ARMSA-Acculturation Scale for Mexican Americans

1. What ethnic identification (does/did) your mother use?

(Note to interviewers: First ask this question and record the response given

to you.) Follow with the following prompts:

- - **What do you think this question is asking?**
  - **What does the term “ethnic identification” mean to you?**

If respondents do not provide an answer or do not understand what you are

asking, follow up this question by asking:

Would you say you are:

Latino,

Hispanic,

American,

North American,

Cuban,

Mexicano, or

Something else? (Specify): ____________

**III. Questions About Friends and Neighbors**

1. When you were growing up, how many of your friends were Hispanic? Would you say….

None of your friends, 1

A few of your friends, 2

Some of your friends, 3

Most of your friends, or 4

All of your friends? 5

- - **What do you think this question asking?**
  - **What does “growing up” mean to you in this question?**
  - **Were you thinking about your time here in the U.S. or in your country?**
  - **What does “Hispanic origin” mean to you?**
  - **What is the difference between “a few of your friends” and “some of your friends”?**

12. When you were growing up, how many of your friends were of Anglo origin? Would you say….

None of your friends, 1

A few of your friends, 2

Some of your friends, 3

Most of your friends, or 4

All of your friends? 5

**What does the word “Anglo” mean to you?** (Note to interviewers: If asked,

- - “Anglo” means “not Hispanic or Latino”)
  - **When you answered this question, what were you thinking about? Growing up in your country? Growing up in the US? Both?**

13. How many of your friends **now** are of Anglo origin? Would you say ….

None of your friends, 1

A few of your friends, 2

Some of your friends, 3

Most of your friends, or 4

All of your friends?.................................. 5

- - **What do you think this question asking?**
  - **How did you interpret the word “now”?**

14. How many of your friends now are of **Hispanic** origin. Would you say…..

None of your friends, 1

A few of your friends, 2

Some of your friends, 3

Most of your friends, or 4

All of your friends?...................... 5

From GAI and LAECA

15. Currently your circle of friends is (Use a card to show respondent the answer choices):

Almost exclusively Hispanics/Latinos (Chicanos/Mexican
 Americans, Puerto Ricans, Cubans, Colombians,
 Dominicans, etc.), 1

Mainly Hispanics/Latinos, 2

Equally Hispanics/Latinos and Americans from the United
 States (Anglo Americans, African Americans,

Asians/Pacific Islanders, etc.), 3

Mainly Americans from the US, or 4

Almost exclusively Americans from the US 5

- - **We’ve given you a lot of answer categories. What did you think each one meant?**
  - **What does “Almost exclusively” mean to you? How is that different from “mainly”?**
  - **What does “circle of friends” mean to you?**

16. Which of the following best describes the people in your neighborhood? Would you say……

All of them are Hispanic,. 1

Most of them are Hispanic, 2

Half of them are Hispanic, 3

Few of them are Hispanic, or 4

None of them are Hispanic? 5

- - **How did you decide on your answer?**
  - **What area were you including as your “neighborhood”?**

**Attitudes About Yourself-** From AMAS

Now, thinking about yourself, how much do you agree or disagree with the next two statements?

17. I think of myself as being U.S. American. Do you….

Strongly agree, 1

Agree somewhat, 2

Disagree somewhat, or 3

Strongly disagree? 4

- - **In your own words, what do you think this question is asking about?**
  - **What does being “U.S. American” mean to you?**

Now, please complete the following statement about what country you usually identify with.

From AMAS

18. I feel good about being U.S. American. Do you….

Strongly agree, 1

Agree somewhat, 2

Disagree somewhat, or 3

Strongly disagree? 4

Now, please complete the following statement about what country you usually identify with.

19a. I think of myself as being _________________________.

(IF DIFFICULTY RESPONDING, PROBE WITH HISPANIC?, LATINO? AND THEN RECORD RESPONSE)

19b. How strongly do you think of yourself as being [19a response]? Would you say…

Very strongly, 1

Strongly 2

Somewhat strongly, or 3

Not very strongly? 4

20. How do you feel about being [19a response]? Would you say you feel …

Very good, 1

Good, 2

Somewhat good, or 3

Not very good? 4

From GAI

21. How proud are you of your Hispanic background? Would you say you are…..

Very proud, 1

Proud, 2

Somewhat proud, 3

Not very proud, or 4

Not proud at all? 5

- **What do you think this question is asking you about?**
- **What do you think being "proud" means?**
- **What does “Hispanic background” mean to you?**
- **Is there another word you prefer to “Hispanic”?**
- **How do you show your pride?**

**V. Questions About Behaviors – From LAECA**

22. How often do you eat Hispanic foods? Would you say…

All the time, 1

Most of the time, 2

Half of the time, 3

Less than half of the time, or 4

Never? 5

- **In your own words, what do you think this question is asking?**
- **How did you come up with your answer?**
- **What types of foods do you consider “Hispanic foods”?**

23. How often do you celebrate in Hispanic tradition? Would you say…

All the time, 1

Most of the time, 2

Half of the time, 3

Less than half of the time, or 4

Never? 5

- **What types of Hispanic traditions were you thinking about?**
- **How did you come up with your answer?**

**Physical Activity Questions**

From NHIS, 2000

The next questions are about physical activities like exercise, sports, or physically active hobbies that you may do in your LEISURE time.

1. How often do you do VIGOROUS leisure-time physical activities for AT LEAST 10 MINUTES that cause HEAVY sweating or LARGE increases in breathing or heart rate? You can answer in times per day, per week, per month, or per year.

* ENTER TIME PERIOD FOR VIGOROUS LEISURE-TIME PHYSICAL ACTIVITIES.

___NEVER

___TIMES PER DAY

___TIMES PER WEEK

___TIMES PER MONTH

___TIMES PER YEAR

___UNABLE TO DO THIS ACTIVITY

- **What does “leisure time” mean to you?**
- **What does “vigorous” mean to you?**
- **What leisure-time physical activities were you thinking about?**
- **How do you know how long you were exercising?**

2. About how long do you do these vigorous leisure-time physical activities each time?

* ENTER TIME PERIOD FOR LENGTH OF VIGOROUS LEISURE-TIME PHYSICAL ACTIVITIES.

|___|___| MINUTES |___|___| HOUR(S)

- **Tell me how you came up with your answer?**
- **How many activities were you thinking about?**
- **How long do you usually do those activities? (the ones you were thinking about)**

3. How often do you do LIGHT OR MODERATE LEISURE-TIME physical activities for AT LEAST 10 MINUTES that cause ONLY LIGHT sweating or a SLIGHT to MODERATE increase in breathing or heart rate? You can answer in times per day, per week, per month, or per year.

* ENTER TIME PERIOD FOR LIGHT OR MODERATE LEISURE-TIME PHYSICAL ACTIVITIES

___NEVER

___TIMES PER DAY

___TIMES PER WEEK

___TIMES PER MONTH

___TIMES PER YEAR

___UNABLE TO DO THIS ACTIVITY

4. About how long do you do these light or moderate leisure-time physical activities each time?

* ENTER TIME PERIOD FOR LIGHT OR MODERATE LEISURE-TIME PHYSICAL ACTIVITIES

|___|___| MINUTES |___|___| HOUR(S)

- **Tell me how you came up with your answer.**

1. How often do you do LEISURE-TIME physical activities specifically designed to STRENGTHEN your muscles, such as lifting weights or doing calisthenics? (Include all such activities even if you have mentioned them before.) You can answer in times per day, per week, per month, or per year.

* ENTER TIME PERIOD FOR STRENGTHENING ACTIVITIES

___NEVER

___TIMES PER DAY

___TIMES PER WEEK

___TIMES PER MONTH

___TIMES PER YEAR

___UNABLE TO DO THIS ACTIVITY

- **(IF R REPORTED A TIME): What activities were you thinking of?**

The next questions are about walking. First I will ask about walking for transportation, that is, walking to get some place. PLEASE INCLUDE ALL WALKS THAT INVOLVED AN ERRAND OR TO GET SOME PLACE. I will ask you separately about walking for other reasons like relaxation or exercise.

1. During the PAST SEVEN DAYS, did you walk to get to some place that took you AT LEAST 10 MINUTES.

*CIRCLE NUMBER FOR RESPONSE

YES 1

NO 2

- **If yes, describe the walk(s) you were thinking about.**
- **How do you remember that the walk(s) took at least 10 minutes?**
- **What days were you thinking about?**

7. During the PAST SEVEN DAYS, ON HOW MANY DAYS did you walk for at least 10 minutes at a time to get some place such as work, school, a store, or restaurant?

*ENTER NUMBER OF DAYS REPORTED

|___|___|

DAY(S)

- **How did you come up with your answer?** (Probe if it is not clear whether the respondent was thinking about one-way trips or two-way trips.)

8. fill1: How much time did you spend walking to get from place to place on that day?

fill2: How much time did you usually spend on one of those days walking to get from place to place?

*ENTER TIME PERIOD FOR LENGTH OF WALKING FOR TRANSPORTATION.

|___|___| MINUTES |___|___| HOUR(S)

9. Sometimes you may walk for fun, relaxation, exercise, or to walk the dog. During the PAST SEVEN

DAYS, DID YOU WALK FOR AT LEAST 10 MINUTES AT A TIME for any of these reasons? Please

do not include any walking that you already told me about.

*CIRCLE NUMBER FOR RESPONSE

YES 1

NO 2

- **If yes. Please describe the walk or walks you were thinking about.**
- **How did you decide if a walk was for fun or exercise instead of a trip to get some place?**
- **How do you remember that the walk or walks took at least 10 minutes?**

1. During the past seven days, on how many days did you walk for at least 10 minutes at a time for fun, relaxation, exercise, or to walk the dog?

*ENTER NUMBER OF DAYS REPORTED

|___|___|

DAY(S)

- **How did you arrive at your answer?**
- **How easy or difficult was it to answer this question? Why?**

11. fill1: How much time did you spend walking on that day for FUN, RELAXATION, OR EXERCISE?

fill2: How much time did you usually spend on one of those days walking for FUN, RELAXATION, OR EXERCISE?

*ENTER TIME PERIOD FOR LENGTH OF WALKING FOR FUN, RELAXATION, OR EXERCISE.

|___|___| MINUTES |___|___| HOUR(S)

12. Which one of the following BEST describes your usual daily activities related to moving around? Do NOT include exercises, sports, or physically active hobbies done in your leisure time.

Do you . . . (READ CATEGORIES AND CIRCLE NUMBER FOR RESPONSE):

Sit during MOST of the day, 1

Stand during MOST of the day, or 2

Walk AROUND MOST of the day? 3

- **Tell me what you think this question is asking.**
- **What types of daily activities were you thinking about?**
- **Take me through a usual day for you.**
- **What does the word “usual” mean to you?**

13. Which one of the following BEST describes your usual daily activities related to lifting or carrying?

things? Do NOT include activities done in your leisure time.

Do you. . . (READ CATEGORIES, USE SHOW CARD, AND CIRCLE NUMBER FOR RESPONSE)

READ IF NECESSARY: Pick the one you do MOST often.

Not lift or carry things very often, 1

Lift or carry LIGHT loads, 2

Lift or carry MODERATE loads, 3

Lift or carry HEAVY loads, or 4

Are you unable to lift or carry loads? 5

OTHER 6

- **When you answered ________ what kinds of things were you thinking about?**

14. fill1: Outside of work, how many hours do you spend per day during **WEEKDAYS** sitting?

14. fill2: How many hours do you spend per day during WEEKDAYS sitting?

*READ IF NECESSARY: Include watching television or videos, working on the computer, playing video games, using the Internet, knitting, sewing, reading, fishing, taking long drives, watching ball games, or doing other sitting activities.

*CHECK APPROPRIATE RESPONSE CATEGORY

___NONE

___1–24 HOUR(S)

- **What do you think this question is asking?**
- **Describe for me how you came up with your answer.**
- **What days were you thinking about?**

15. fill1 Outside of work, how many hours do you spend per day during the **WEEKEND** sitting?

fill2: How may hours do you spend during the WEEKEND sitting?

*READ IF NECESSARY: Include watching television or videos, working on the computer, playing video games, using the Internet, knitting, sewing, reading, fishing, taking long drives, watching ball games or doing other sitting activities. Weekend means any days off, not necessarily Saturday and Sunday.

*CHECK APPROPRIATE RESPONSE CATEGORY

___NONE

___1–24 HOUR(S)

- **What do you think this question is asking?**
- **How easy or difficult was it for you to answer this question? Can you say more about that?**

16. During the PAST 12 MONTHS, did a doctor or other health professional RECOMMEND that you BEGIN or CONTINUE to do any type of exercise or physical activity?

*CIRCLE NUMBER FOR RESPONSE

YES 1

NO 2

DID NOT SEE A DOCTOR IN THE **PAST**

**12 MONTHS** 3

REFUSED 7

DK 8

- **What is this question asking?**
- **(If YES): Who recommended it to you? A doctor, Someone else? Who?**
- **(If YES): What did he (she) say and when did he (she) say it?**

FINAL ACCULTURATION/PHYSICAL ACTIVITY QUESTIONS-ROUND 1

**Preguntas sobre Aculturación**

Introducción: Hoy le preguntaremos sobre dos temas principales-la aculturación y la actividad física. Al decir aculturación, queremos decir al grado al que usted ha adoptado a la cultura norteamericana estadounidense o anglo. Las preguntas sobre la actividad física se enfocarán en cosas como el ejercicio y el caminar. Ahora, empecemos.

**I. Idioma**

1. Por lo general, ¿qué idioma *habla* usted? Usted diría… (Lea las categorías y marque con un círculo el número que corresponda a su respuesta.

Solamente español………………………………………..1

Más en español que inglés, ………………………………2

Igual en español y en inglés (bilingüe)………………….3

Más en inglés que español, ……………………………...4

Solamente inglés…u……………………………………….5

Otro idioma?.................................................................6 Especifique-----------------------------------

Prefiere no contestar…………………….…………………7

No sabe………………………………………………………8

- ¿Por qué respondió así (¿Cómo decidió que habla usted en _________ más ?)

- ¿Las opciones presentadas corresponden a su situación? Si no, por favor diga porque.

2. ¿Qué idioma habló cuando era niño(a)? Diría usted …

Solamente español……………………………………..1

Más en español que inglés………………………………2

Igual en español y en inglés (bilingüe)………………….3

Más en inglés que español, ……..……………………...4

Solamente inglés…u………………….………………….5

Otro idioma?.................................................................6 Especifique-----------------------------------

Prefiere no contestar………………………..……………7

No sabe……………………………………………………8

- ¿Por qué respondió así? (¿Cómo decidió que hablaba usted en _________ más ?)
- ¿Qué significa para usted la frase “cuando era niño”?

3. Por lo general,¿qué idioma *lee* usted mejor? ¿Diría usted …

Solamente español………………………………………..1

Más en español que inglés ………………………………2

Igual en español y en inglés (bilingüe)………………….3

Más en inglés que español, ………………………………...4

Solamente inglés u………………………………………….5

Otro idioma?.................................................................6 Especifique-----------------------------------

Prefiere no contestar………………………………………7

No sabe……………………………………………………8

- ¿Por qué contesto así ? (¿Cómo decidió que lee usted _________ mejor?)
- ¿En que tipos de lectura o material para leer estaba usted pensando?

4. ¿ Qué idioma habla más en casa? ¿Diría…

Solamente español………………………………………..1

Más el español que inglés, ………………………………2

Igual en español y en inglés (bilingüe)………………….3

Más en inglés que español, …………………..………....4

Solamente inglés…u.…………………………….……….5

Otro idioma?..................................................................6 Especifique----------------------------------

Prefiere no contestar………………………………………7

No sabe…………………………………………………..…8

- ¿ Por que respondió así? (¿Cómo decidió que habla usted _________ más ?)

5. ¿Qué idioma habla más con sus amigos? ¿Diría…

Solamente español………………………………………..1

Más en español que inglés…. ………………..…………2

Igual en español y en inglés (bilingüe)………………….3

Más en inglés que español, ..……………………………...4

Solamente inglés…u……………………………………….5

Otro idioma?.................................................................6 Especifique-----------------------------------

Prefiere no contestar………………………………………7

No sabe……………………………………………………8

- ¿Por qué respondió así? (¿Cómo decidió que habla usted _________ más ?)

6. ¿En que idioma piensa usted más ? ¿Usted diría…

Solamente español………………………………………..1

Más en español que inglés………………………………2

Igual en español y en inglés (bilingüe)………………….3

Más en inglés que español……………………………...4

Solamente inglés……u…………………………………….5

Otro idioma?.................................................................6 Especifique-----------------------------------

Prefiere no contestar………………………………………7

No sabe……………………………………………………8

- ¿Por qué respondió así? (¿Cómo decidió que piensa usted en _________ más ?)

7. ¿ En qué idioma son los programas de televisión que usted usualmente ve? ¿Diría…

Solamente español………………………………………..1

Más en español que inglés………………………………2

Igual en español y en inglés (bilingüe)………………….3

Más en inglés que español……………………………...4

Solamente inglés…u……………………………………….5

Otro idioma?.................................................................6 Especifique-----------------------------------

Prefiere no contestar………………………………………7

No sabe……………………………………………………8

- ¿Por qué respondió así? (¿Cómo decidió que usted ve programas en _________ más ?)
- ¿Que tipos de programas de televisión ve usted?

8. ¿ En qué idioma son los programas de radio que usted usualmente escucha? ¿Diría…

Solamente español……………………………………..1

Más en español que inglés………………………………2

Igual en español y en inglés (bilingüe)………………….3

Más en inglés que español……………………………...4

Solamente inglés……u………………………………….5

Otro idioma?.................................................................6 Especifique-----------------------------------

Prefiere no contestar………………………………………7

No sabe……………………………………………………8

- Le acabamos de hacer unas cuantas preguntas acerca de idioma. ¿Qué tan difícil o fácil le resultó contestar?

**II. Información Sobre sus Padres, Abuelos, y Usted**

9.a ¿ Dónde nació su mamá? (Respondents will report an answer, if they can, and the interviewer will then circle the NUMBER FOR THE response).

Estados Unidos 1

Mexico 2

Cuba 3

Puerto Rico 4

***(protocol should follow up***

***on which country)***

Centro America (Nombre del país) .. 5

Sud America (Nombre del país) … …. 6

Otro 7

No sabe……….………………………………8

9.b ¿Dónde nació su papá?

Estados Unidos 1

Mexico 2

Cuba 3

Puerto Rico 4

***(protocol should follow up***

***on which country)***

CENTRO AMERICA (Nombre el país) … 5

Sud America (Nombre el país) … …. 6

Otro 7

No sabe……….………………………………8

9.c ¿ Dónde nació el padre de su mamá?

Estados Unidos 1

Mexico 2

Cuba 3

Puerto Rico 4

***(protocol should follow up***

***on which country)***

Centro America (Nombre el país) … 5

Sud America (Nombre el país) … …. 6

Otro 7

No sabe……….………………………………8

- ¿Acerca de quien en su familia estamos preguntando?

9d. ¿ Dónde nació el padre de su papá?

Estados Unidos 1

Mexico 2

Cuba 3

Puerto Rico 4

***(protocol should follow up***

***on which country)***

Centro America (Nombre del país) … 5

Sud America (Nombre del país) ….. … 6

Otro 7

No sabe……….………………………………8

- ¿Acerca de quién en su familia estamos preguntando?

9.e ¿ Dónde nació la madre de su mamá?

Estados Unidos 1

Mexico 2

Cuba 3

Puerto Rico 4

***(protocol should follow up***

***on which country)***

Centro America (Nombre del país) 5

Sud America (Nombre del país) … …. 6

Otro 7

No sabe……….………………………………8

9.f ¿ Dónde nació la madre de su papá?

Estados Unidos 1

Mexico 2

Cuba 3

Puerto Rico 4

***(protocol should follow up***

***on which country)***

Centro America (Nombre el país) … 5

Sud America (Nombre el país) … …. 6

Otro 7

No sabe……….………………………………8

- ¿Que tan fácil o difícil le resulto contestar a estas preguntas acerca de sus abuelos?

10. ¿Cuál identificación étnica usa (usaba) su mamá?

(Note: Primero los entrevistadores preguntan y anotan la respuesta) siguiendo las siguientes preguntas:

- ¿Que es lo que se esta preguntando aquí?
- Que significa la frase “identificación étnica”, para usted?

(Si los entrevistados no responden o no entienden la pregunta, continué preguntando.

¿Diría que ella es/era…:

Latina,

Hispana,

Americana,

Norte Americana,

Cubana,

Mexicana, o

Algún otro grupo étnico? (Especifique): ____________

**III. Preguntas Sobre Amigos y Vecinos**

11. Durante su niñez o juventud, ¿cuántos de sus amigos eran hispanos?

Ninguno de sus amigos, 1

Pocos de sus amigos, 2

Algunos de sus amigos, 3

La mayoría de sus amigos 4

Todos sus amigos 5

- ¿Que es lo que se esta preguntando aquí?
- ¿Que significa para usted la frase “durante su niñez o juventud” en esta pregunta?
- ¿Estaba usted pensando en su tiempo aquí en los Estados Unidos o en su país?
- ¿Qué significa para usted la frase “de origen hispano”?
- ¿Cuál es la diferencia entre “pocos de sus amigos” y “alguno de sus amigos”
- ¿Cuál palabra tiene más sentido para Ud.? “hispano” o “latino”

12. Durante su niñez o juventud, ¿cuántos de sus amigos eran de origen Anglo?

Ninguno de sus amigos, 1

Pocos de sus amigos, 2

Algunos de sus amigos, 3

La mayoría de sus amigos 4

Todos sus amigos 5

- ¿Que significa para usted “Anglo”?
- Cuando usted respondió esta pregunta, ¿en que estaba pensando? ¿Su niñez o juventud en su país, en los Estados Unidos o ambos?

13. Actualmente, ¿cuántos de sus amigos son anglos? ¿Diría que…

Ninguno de sus amigos, 1

Pocos de sus amigos, 2

Alguno de sus amigos, 3

La mayoría de sus amigos 4

Todos sus amigos 5

- ¿Que es lo que se esta preguntando aquí?
- ¿Cómo interpreta la frase “amigos actuales”?

14. ¿Cuántos de sus amigos son de origen **hispano** actualmente? ¿Diría que…

Ninguno de sus amigos, 1

Pocos de sus amigos, 2

Alguno de sus amigos, 3

La mayoría de sus amigos 4

Todos sus amigos 5

15. Actualmente su círculo de amigos es: (Use a card to show respondent the answer choices):

Casi exclusivamente Hispano/Latino (chicanos/mexicanos americanos, puertorriqueños, cubanos, colombianos, dominicanos, etc. 1

Por la mayoría hispanos/Latinos, 2

Casi igual hispanos/latinos y americanos de los Estados Unidos (Anglo Americanos, Africanos Americanos,

Asiáticos/de la Isla del Pacifico, etc.), 3

Por la mayoría de los EEUU o 4

Casi exclusivamente americanos de los EEUU 5

- Le hemos ofrecido muchas opciones como respuestas. ¿Qué cree que significa cada uno?
- ¿Usted prefiere la palabra Hispano(a) o Latino (a)? ¿Por qué?
- ¿Que significa para usted “casi exclusivamente”?
- ¿Cómo es “casi exclusivamente” diferente a “en su mayoría” ?
- ¿Qué significa “circulo de amigos” para usted”?

16. ¿Cuál de las siguientes frases mejor describe la gente de su vecindad ? Diría…

Todos son hispanos 1

La mayoría son hispanos, 2

La mitad son hispanos, 3

Pocos son hispanos

Ningunos son hispano 5

- ¿Como decidió contestar así?
- ¿Qué área incluyó usted como su “vecindad ”?

**IV. Acerca de uno mismo**

Ahora, vamos a pensar acerca de usted mismo, ¿ está de acuerdo o no con las siguientes frases?

17. Yo me considero americano estadounidense. Usted esta…

totalmente de acuerdo, 1

un poco acuerdo, 2

un poco en desacuerdo 3

totalmente en desacuerdo 4

- En sus propias palabras, ¿que piensa, que es lo que esta preguntando?
- ¿Qué es lo que significa ser “americano estadounidense” para usted?

18. Me siento bien ser norteamericano u americano estadounidense.

¿Usted esta...

totalmente de acuerdo, 1

un poco acuerdo, 2

un poco en desacuerdo 3

totalmente en desacuerdo 4

Ahora, Por favor complete la siguiente frase acerca del país con que más se identifica usted.

19 a. Yo me considero ser ________________________

(¿Si es difícil responder esta pregunta, trate con la palabra hispano?, ¿latino? Y luego anote la respuesta.

19b. ¿Qué tan fuerte es su identificación con ser [respuesta 19a]? ¿Usted diría…

Muy fuerte, 1

Fuerte 2

Algo fuerte o 3

No muy fuerte? 4

20. ¿Cómo se siente acerca de ser [respuesta 19a]? ¿Usted diría que se siente…

Muy bien, 1

Bien, 2

Más o menos bien, o 3

No muy bien 4

21. ¿Qué tan orgulloso es usted de su origen hispano? ¿Diría que…

Muy orgulloso, 1

Orgulloso, 2

Algo orgulloso, 3

No muy orgulloso, o 4

Nada orgulloso(a)? 5

- ¿Qué es lo que se esta preguntando aquí?
- ¿Qué significa ser “orgulloso” para usted?
- ¿Qué significa “de origen hispano” para usted?
- ¿Hay alguna otra palabra que prefiera usar en vez de “Hispano”?
- Como demuestra su orgullo?

**V. Preguntas acerca de comportamientos**

22. ¿Con qué frecuencia come usted comida hispana?

Todo el tiempo, 1

Casi todo el tiempo, 2

La mitad del tiempo, 3

Menos de la mitad del tiempo 4

Nunca? 5

- Con sus propias palabras, ¿qué es lo que esta pregunta esta preguntando? ¿A que se refiere esta pregunta?
- ¿Como llegó a esta respuesta?
- ¿Qué tipo de comidas considera usted “comidas hispanas”?

23. ¿Con qué frecuencia celebra usted tradiciones hispanas? ¿Usted diría…

Todo el tiempo, 1

Casi todo el tiempo, 2

La mitad del tiempo, 3

Menos de la mitad del tiempo, o 4

Nunca? 5

- ¿De que tipo de tradiciones hispanas estaba usted pensando?
- ¿Cómo llego a la respuesta?

**Preguntas sobre la Actividad Física**

Las siguientes preguntas se tratan de actividades físicas como el ejercicio, deportes, o aficiones físicamente activas que usted puede hacer en su TIEMPO LIBRE.

1. ¿Con qué frecuencia hace actividades FUERTES O VIGOROSAS en su tiempo libre DE POR LO MENOS 10 MINUTOS que lo hacen sudar MUCHO, o aumentan BASTANTE su respiración o ritmo cardíaco? Puede contestar con el número de veces al día, a la semana, al mes, o al año.

___NEVER

___TIMES PER DAY

___TIMES PER WEEK

___TIMES PER MONTH

___TIMES PER YEAR

___UNABLE TO DO THIS ACTIVITY

- ¿Qué significa para usted, la frase “tiempo libre”?
- ¿Qué significa para usted, la palabra “ fuertes o vigorosas”?
- ¿En que tipo de actividades físicas que hace durante su tiempo libre estaba pensando?
- ¿Cómo sabe cuanto tiempo estuvo haciendo esta actividad física?

2. ¿Por cuánto tiempo hace estas actividades fuertes o vigorosas en su tiempo libre cada vez?

* ENTER TIME PERIOD FOR LENGTH OF VIGOROUS LEISURE-TIME PHYSICAL ACTIVITIES.

|___|___| MINUTES |___|___| HOUR(S)

- Dígame por favor, como llegó a esta respuesta.
- ¿Cuántas actividades incluyó en su respuesta?
- ¿Cuánto tiempo suele hacer cada una de estas actividades físicas?

3. ¿Con qué frecuencia HACE actividades físicas LIGERAS/LIVIANAS A MODERADAS EN SU TIEMPO LIBRE DE POR LO MENOS 10 MINUTOS que lo hacen sudar UN POCO o aumentan LEVEMENTE su respiración o ritmo cardíaco? Puede contestar con el número de veces al día, a la semana, al mes, o al año.

* ENTER TIME PERIOD FOR LIGHT OR MODERATE LEISURE-TIME PHYSICAL ACTIVITIES

___NEVER

___TIMES PER DAY

___TIMES PER WEEK

___TIMES PER MONTH

___TIMES PER YEAR

___UNABLE TO DO THIS ACTIVITY

- ¿Qué significa la palabra liviana/ ligera?

4. ¿Por cuánto tiempo hace estas actividades ligeras/livianas a moderadas en su tiempo libre cada vez?

* ENTER TIME PERIOD FOR LIGHT OR MODERATE LEISURE-TIME PHYSICAL ACTIVITIES

|___|___| MINUTES |___|___| HOUR(S)

- Dígame por favor, cómo llegó a este número.

5. ¿Con qué frecuencia hace actividades físicas EN SU TIEMPO LIBRE específicamente para FORTALECER sus músculos, tal como levantar pesas o ejercicios calisténicos? (Incluya todas las actividades aunque las haya mencionado anteriormente.) Puede contestar con el número de veces día, a la semana, al mes, o al año.

* ENTER TIME PERIOD FOR STRENGTHENING ACTIVITIES

___NEVER

___TIMES PER DAY

___TIMES PER WEEK

___TIMES PER MONTH

___TIMES PER YEAR

___UNABLE TO DO THIS ACTIVITY

- (If R reported a time) ¿En qué o cuál tipo de actividades estaba pensando?

Las siguientes preguntas son acerca de caminar. Primero, le preguntaré sobre caminar para trasladarse, o sea, caminar de un sitio a otro. POR FAVOR INCLUYA EL CAMINAR YA SEA PARA ALGUN MANDADO O PARA LLEGAR A ALGUN SITIO. Después le preguntaré acerca de caminar por otras razones como relajamiento o ejercicio.

6. Durante los ULTIMOS SIETE DIAS, ¿caminó usted por POR LO MENOS 10 MINUTOS para llegar a algún sitio?

1. Sí

2. No

- Si R contesta “Sí”- Por favor platíqueme acerca de estas caminatas.
- ¿Cómo recuerda que esta(s) caminatas duraron por lo menos 10 minutos?
- ¿Hace cuánto tiempo fue (ron) esta(s) caminatas?

7. Durante LOS ULTIMOS SIETE DIAS, ¿ CUANTOS DIAS caminó usted por lo menos 10 minutos a la vez para llegar a algún lugar tal como el trabajo, la escuela, una tienda o un restaurante?

*ENTER NUMBER OF DAYS REPORTED

|___|___|

DAY(S)

- ¿Cómo llegó usted a este número?

(Probe if it is not clear whether the respondent was thinking about one-way trips or two-way trips.)

8. fill1: ¿Cuánto tiempo se tardó en caminar para llegar de un lugar a otro en ese día?

fill2: En uno de esos días, ¿cuánto tiempo usualmente se tardó caminando para llegar de un sitio a otro?

*ENTER TIME PERIOD FOR LENGTH OF WALKING FOR TRANSPORTATION.

|___|___| MINUTES |___|___| HOUR(S)

9. A veces usted puede caminar para diversión, relajamiento, ejercicio o para sacar al perro. Durante los ULTIMOS SIETE DIAS, ¿CAMINO USTED POR LO MENOS DIEZ MINUTOS A LA VEZ por alguna de estas razones? Por favor no incluya las veces que ya ha mencionado.

1. Sí

2. No

- Si R contesta “Sí”- Por favor platíqueme acerca de esta(s) caminata(s).
- ¿Cómo decidió que esta caminata era para diversión o ejercicio en vez de una forma de llegar a un lado de otro?
- ¿Cómo recuerda que esta(s) caminata(s) duraron por lo menos 10 minutos?

10. Durante los últimos siete días, ¿cuántos días caminó usted por lo menos diez minutos a la vez para diversión, relajamiento, ejercicio o para sacar al perro?

*ENTER NUMBER OF DAYS REPORTED

|___|___|

DAY(S)

- Dígame, por que contesto así?
- ¿Qué tan fácil o difícil le resulto contestar esta pregunta?

11. fill1: ¿Cuánto tiempo pasó usted caminando en ese día para DIVERSION, RELAJAMIENTO, O EJERCICIO?

Fill2: ¿Cuánto tiempo pasó usted usualmente en uno de esos días caminando para DIVERSION, RELAJAMIENTO O EJERCICIO?

*ENTER TIME PERIOD FOR LENGTH OF WALKING FOR FUN, RELAXATION, OR EXERCISE.

|___|___| MINUTES |___|___| HOUR(S)

12. ¿Cuál de estas frases MEJOR caracteriza sus actividades diarias en relación con moverse? NO INCLUYA ejercicios, deportes o pasatiempos físicos realizados en su tiempo libre.

¿Usted… (read categories below):

1. ¿Está SENTADO(A) durante la MAYOR parte del día?

2. ¿Se mantiene DE PIE durante la MAYOR parte del día?

3. ¿CAMINA durante la MAYOR parte del día?

- ¿Qué es lo que se esta preguntando aquí?
- ¿En que tipo de “actividades diarias” estaba usted pensando?
- Platíqueme de un día típico para usted.
- ¿Qué significa la palabra “pasatiempos”? ¿Cómo es semejante/diferente a la palabra “aficiones” que se usó en una pregunta anterior? ¿Cuál palabra cree que es mejor?

13. ¿Cuál de estas frases MEJOR caracteriza sus actividades diarias en relación con levantar o cargar objetos? NO incluya actividades hechas en su tiempo libre.

¿Usted… (read categories, USE SHOW CARD AND CIRCLE NUMBER FOR RESPONSE) 1-4 below):

*Read if necessary: Seleccione lo que hace con MÁS frecuencia.

1. ¿NO levanta o carga objetos muy frecuentemente?

2. ¿Levanta cargas LIVIANAS?

3. ¿Levanta cargas MODERADAS?

4. ¿Levanta cargas PESADAS?

5. No puede levantar o acarrear cargas

6. Otra?

- ¿De que tipos de cosas (cargas) estaba pensando cuando contestó ______________?

14. Fill1: Fuera del trabajo, ¿cuántas horas al día se pasa sentado(a) DURANTE LA SEMANA LABORAL?

14. Fill2: ¿Cuántas horas al día se pasa sentado(a) DURANTE LA SEMANA LABORAL?

*Read if necessary: Incluya ver televisión o videos, trabajar con la computadora, jugar con juegos de video, usar la Internet, tejer, coser, leer, pescar, viajes largos en carro, ver deportes, o hacer otras actividades sentado.

*CHECK APPROPRIATE RESPONSE CATEGORY

___NONE

___1–24 HOUR(S)

- ¿Qué es lo que se esta preguntando aquí?
- Por favor, dígame cómo es que llegó a su respuesta.
- ¿En que días estaba pensando cuando contestó usted esta pregunta?
- ¿Qué significa la frase “semana laboral” para usted? ¿Qué días incluye la semana laboral?

15. fill1: Fuera del trabajo, ¿cuántas horas al día se pasa sentado(a) durante **EL FIN DE SEMANA**?

Fill2: ¿Cuántas horas se pasa sentado(a) durante **EL FIN DE SEMANA?**

*Read if necessary: Incluya ver televisión o videos, trabajar con la computadora, jugar con juegos de video, usar la Internet, tejer, coser, leer, pescar, viajes largos en carro, ver deportes, o hacer otras actividades sentado. El fin de semana significa los días cuando no trabaja y puede ser otros días de sábado y domingo.

*CHECK APPROPRIATE RESPONSE CATEGORY

___NONE

___1–24 HOUR(S)

- ¿Qué es lo que se esta preguntando aquí?
- ¿Se le hizo fácil o difícil contestar esta pregunta? ¿Me podría explicar porque se le hizo fácil/difícil?
- ¿En que días estaba pensando cuando contestó usted esta pregunta?

16. Durante LOS ULTIMOS 12 MESES, ¿algún médico u otro profesional de la salud le ha RECOMENDADO que EMPIEZE o CONTINUE haciendo ejercicios o actividades físicas?

*CIRCLE NUMBER FOR RESPONSE

YES 1

NO 2

DID NOT SEE A DOCTOR IN THE **PAST**

**12 MONTHS** 3

REFUSED 7

DK 8

- ¿Qué es lo que se esta preguntando aquí?
- (If YES): ¿Quién se lo recomendó? ¿Fue un médico, otra persona,…?
- (If YES): ¿Qué dijo? ¿Cuándo?
